# Post-Transcriptional Control of Gene Expression in Mouse Early Embryo Development: A View from the Tip of the Iceberg

**DOI:** 10.3390/genes2020345

**Published:** 2011-04-06

**Authors:** Enrica Bianchi, Claudio Sette

**Affiliations:** 1 Department of Public Health and Cell Biology, Section of Anatomy, University of Rome “Tor Vergata”, 00133 Rome, Italy; E-Mail: enrica.bianchi@uniroma2.it; 2 Laboratory of Neuroembryology, Fondazione Santa Lucia IRCCS, 00143 Rome, Italy

**Keywords:** fertilization, early embryogenesis, meiosis, maternal factors, RNA metabolism, mRNA translation, genome activation

## Abstract

Fertilization is a very complex biological process that requires the perfect cooperation between two highly specialized cells: the male and female gametes. The oocyte provides the physical space where this process takes place, most of the energetic need, and half of the genetic contribution. The spermatozoon mostly contributes the other half of the chromosomes and it is specialized to reach and to penetrate the oocyte. Notably, the mouse oocyte and early embryo are transcriptionally inactive. Hence, they fully depend on the maternal mRNAs and proteins stored during oocyte maturation to drive the onset of development. The new embryo develops autonomously around the four-cell stage, when maternal supplies are exhausted and the zygotic genome is activated in mice. This oocyte-to-embryo transition needs an efficient and tightly regulated translation of the maternally-inherited mRNAs, which likely contributes to embryonic genome activation. Full understanding of post-transcriptional regulation of gene expression in early embryos is crucial to understand the reprogramming of the embryonic genome, it might help driving reprogramming of stem cells *in vitro* and will likely improve *in vitro* culturing of mammalian embryos for assisted reproduction. Nevertheless, the knowledge of the mechanism(s) underlying this fundamental step in embryogenesis is still scarce, especially if compared to other model organisms. We will review here the current knowledge on the post-transcriptional control of gene expression in mouse early embryos and discuss some of the unanswered questions concerning this fascinating field of biology.

## 1. Introduction: From the Gametes to the Embryo

Oocytes and spermatozoa are terminally differentiated haploid cells which can no longer divide. Both gametes are shaped and programmed to fuse with each other as they touch. In just few hours after fertilization, the haploid parental pronuclei become clearly visible inside the mouse zygote (one-cell embryo) [1]. Before syngamy occurs, both pronuclei replicate their entire DNA, and nearly 20 hours after fertilization the first mitotic division takes place. Thereafter, each blastomere of the newly formed two-cell embryo will have inherited a diploid zygotic nucleus, including a set of paternal and maternal chromosomes [1].

**Figure 1:**
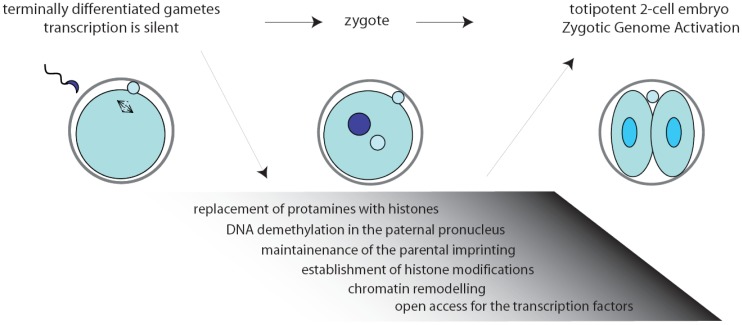
Schematic representation of the changes occurring in the chromatin of the zygote. The blastomeres of the two-cell embryo are the first truly totipotent cells. They originate from two transcriptionally repressed cells and acquire their peculiar capacity through the changes occurring in the zygote. The main necessary modifications concerning the remodeling of the chromatin of the two parental genomes are: erasure of the epigenetic marks that typify the genome of gametes; maintenance of epigenetic marks in the imprinted genes. This extensive reprogramming is carried out by the maternal factors stored in the oocyte.

Inside the environment provided by the oocyte, a complex series of remarkable tasks has to be accomplished rapidly and precisely. First, fertilization triggers the completion of the II meiotic division (MII) of the oocyte. Then, the zygote must resume the mitotic cell cycle, remodel the chromatin, activate transcription, and initiate the embryonic developmental program [2,3] (Figure 1). Noteworthy, the mouse gametes are transcriptionally quiescent at the time of fertilization, and the sperm contribution is mainly represented by its DNA content. As a consequence, the zygote must use pre-existing factors, particularly maternal RNAs and proteins, that had been deposited during oogenesis in the egg cytoplasm [4].

The two-cell embryo stage marks the transition from maternal-to-zygotic gene dependence [4,5]. In fact, activation of transcription of the embryonic genome, usually known as zygotic genome activation (ZGA), progressively frees the embryo from the need to use maternally stored RNAs (Figure 1). In the mouse, an early phase, called minor ZGA or ZGA I, occurs before the first mitosis whereas a second phase, named major ZGA or ZGA II, begins soon after the first mitotic division [3,5–7]. Notably, ZGA I occurs regardless of whether the zygotic S-phase has been completed, but the RNAs transcribed will not be translated until the two-cell stage. On the other side, ZGA II requires the completion of the first cell cycle and accounts for the bulk of both transcription and translation of the embryonic genes [7]. Later on, the embryo will exploit its own transcriptional and translational machinery to carry out its developmental program.

Fertilization creates totipotent cells capable of generating all tissues of the new organism. In this respect, the one-cell embryo can be considered a totipotent cell. However, since the mouse zygote is essentially a transient state in which a diploid nucleus is never formed, the first totipotent cells originated by fertilization are the blastomeres of the two-cell embryo. As embryonic development proceeds, this capability will be progressively reduced as a consequence of the increasing differentiation of the daughter cells into specific cell types. At the blastocyst stage, totipotency will be restricted to pluripotency, but the exact time when this boundary arises is still controversial [8–10]. For instance, it has been recently proposed that the blastomeres of a four-cell embryo can already be distinguished for their fate by histone marks that modify their genome epigenetically [11]. The difference between toti- and pluripotency resides in the ability of the cells to organize into an embryo, which is missed by pluripotent cells. These cells can generate the three germ layers of the embryo, but, unlike totipotent cells, cannot give rise to extra-embryonic tissues. Usually the blastomeres are considered totipotent until the eight-cell stage [12,13] when the first events of morphological differentiation occurs at the morula stage, and the polarized blastomeres generate inner and outer cells by asymmetrical divisions [14]. Noteworthy, early mouse development from the zygote to the morula stage is not accompanied by overall growth in size of the embryo, suggesting that totipotency might be related to the maternally inherited cytoplasmic content [15].

In this review we will focus our attention on one peculiar aspect of early embryonic development: the post-transcriptional control of gene expression. This step in gene expression is essential for the zygote and it is likely to be relevant for the correct onset of the embryonic developmental program. In particular, we will underline the strict connection between a finely tuned translation regulation, chromatin remodeling and zygote genome activation in the mouse embryo. We will also discuss some intriguing new findings about the intracellular localization of maternal mRNAs in mouse.

## 2. Fertilization Triggers Changes in Structure of the Embryonic Chromatin

Chromatin is thought to be the principal controller of the access of the transcription machinery to the mammalian genome. Chromatin structure is organized in nucleosomes, wherein double-stranded DNA is wrapped around histones, and higher-ordered structures obtained by folding of the chromosomes [16]. Genome accessibility is modulated by several modifications that affect the chromatin, such as DNA methylation, covalent modification of histones (acetylation, methylation, sumoylation) and histone replacement through incorporation of the so-called histone variants [17,18]. Altogether these modifications are referred to as epigenetic changes, because they do not alter the genotype but have an impact on the coding potential of the genome and, thereby, the phenotype of the organism.

Chromatin modifications play an essential role during embryonic development, permitting different cells and tissues to progressively acquire specific patterns of gene expression [19]. Extensive remodeling of the chromatin is necessary already at the onset of development, when the zygote needs to remove the epigenetic marks in the oocyte and sperm genomes to convert the highly specialized gene expression signature of the gametes into that of a totipotent early blastomere [19–21]. Noteworthy, all the chromatin modifications in the zygote are carried out while transcription is silenced (Figure 1). Since the proteins required to exert this task would probably interfere with regulation of the parental genomes, it is likely that they are produced after fertilization through orchestration of translational mechanisms.

Upon fertilization, the highly condensed paternal chromatin is rapidly reorganized in order to acquire the appropriate epigenetic state. First, protamines are replaced by histones, followed by active DNA demethylation of the chromatin in the paternal pronucleus that will be completed before DNA replication begins [22,23]. Thus, genes that are highly methylated in the sperm are demethylated in the zygote rapidly after fertilization, while the maternal DNA is preserved from this reaction [24] and the oocyte-derived maternal alleles are unaffected [22]. Only later, during the second and third cleavage stages, the maternal genome will be gradually demethylated by a replication-dependent mechanism [23]. At the same time, amidst the tumultuous events that rapidly occur in the embryonic genome, the imprinted genes are preserved unaltered since they are essential for the formation of a viable zygote [25,26] (Figure 1). The initial difference in chromatin structure between male and female pronuclei may also explain the preferential binding of transcription factors to paternal chromatin observed at the early stages of embryonic development [27,28].

## 3. Maternally Inherited Factors Trigger Epigenetic Reprogramming

**Figure 2:**
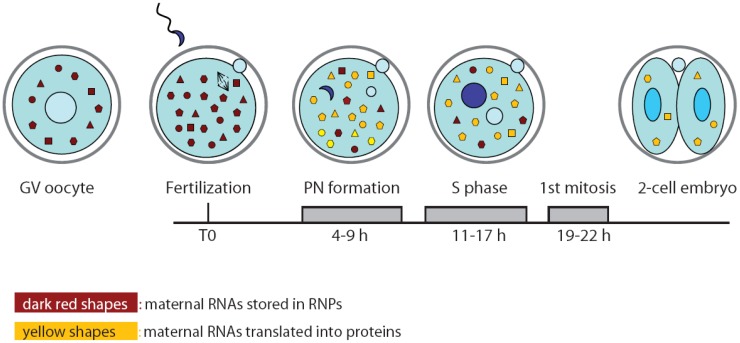
Maternal mRNAs are accumulated during oogenesis and bound by proteins in complexes called RNPs (ribonucleoproteins). Maternal mRNAs are stored in an inactive masked state until recruited for translation in a stage-specific manner. Soon after fertilization maternal RNAs are loaded onto polysomes for translation, in order to provide essential factors to the newly formed zygote, before the full activation of its genome occurs. At the two-cell stage 90% of the maternal transcripts will be degraded. The starting point for timing (hours) is fertilization.

Recently, a novel role for sperm epigenetic marks has been proposed in the establishment of embryonic totipotency [29]. Cairns and colleagues showed that although most of the histones are replaced by protamines in the sperm nucleus, the remaining nucleosomes are wrapped around loci of developmental importance, such as imprinted genes, microRNA and HOX gene clusters. Moreover, this study highlighted a differential representation of specific histone marks in promoters that are activated earlier or later in embryo development [29]. Thus, the epigenetic status of the chromatin in the male pronucleus might be predisposed to insure timely activation of particular developmental loci after ZGA. Nevertheless, such re-activation of gene transcription also requires extensive remodeling of the chromatin by oocyte-inherited maternal factors [30] (Figure 2). For instance, nucleoplasmin 2 (NPM2) is a maternal protein that contributes to nuclear and nucleolar organization of the chromatin. Sperm DNA decondensation proceeds normally without NPM2, however, the oocyte and early embryo nuclei showed several defects in structure and histone marks that strongly impaired development [31]. Similarly, it was shown that the SWI/SNF complex component BRG1 (gene symbol: *Smarca4*) is required for histone methylation and for ZGA in the two-cell embryo [32]. Importantly, the BRG1-interacting protein BRWD1 (bromodomain and WD repeat domain containing 1) is also required for efficient oocyte maturation and early embryonic development [33]. Chromatin remodelers might be attracted to specific loci in the early embryo by transcription factors, such as TIF1α, which translocates from the cytoplasm to the pronuclei and modulates the first wave of embryonic genome activation at ZGA [34].

Maternally-inherited proteins have also been linked to maintenance of the parental imprinting in the early embryo. STELLA (gene symbol: *Dppa3*, developmental pluripotency-associated 3) is an oocyte-specific maternal-effect protein. STELLA is required to maintain methylation marks on most of the maternal genome [35], thereby playing a crucial role in early development [36,37]. An essential function in the maintenance of DNA methylation at imprinted loci during preimplantation development is played by the DNA methyltransferase DNMT1 [38]. On the other hand, the zinc-finger protein ZFP57 is required for the *de novo* establishment of germline methylation, and also plays a broader role in maintaining maternal and paternal methylation patterns in early embryos [39]. Among the maternal factors necessary for ZGA, or implied in the transition from oocyte to embryo, ZAR1 seems to function at the earliest steps. In *Zar1* knockout zygotes pronuclei form and DNA replication starts, but the maternal and paternal genomes remain separate for unknown reasons [40]. The ZAR1 homologue ZAR1L might also play a role in chromatin remodeling and regulation of gene expression in the early embryo [41]. Notably, ZAR1L was shown to colocalize with some components of the RNA granules (P bodies and chromatoid bodies), which may suggest an additional role of this protein in mRNA stability or translation.

## 4. Translational Regulation of Gene Expression in the Mouse Early Embryo

Since *de novo* transcription does not occur during oocyte maturation and the first cell cycle of the embryo, it is likely that the proteins required for the general reprogramming of the cell are translated from pre-existing mRNAs during this developmental window (Figure 2). In particular, the zygote needs to switch from the meiotic to mitotic divisions and to reprogram the haploid specialized genome of the gametes into a totipotent diploid genome. In spite of the importance of this question, only few experiments addressing it have been published to date. Likely, investigators are discouraged by the paucity of material available and the consequent difficulties arising from applying bio-molecular techniques on limited quantities. Nevertheless, a study performed by Latham and colleagues attempted to investigate translation regulation at the onset of embryogenesis by monitoring the recruitment of maternal mRNAs onto the polysomes after fertilization in mouse zygotes. Oocyte and embryo extracts were centrifuged on sucrose gradients to separate the free ribonucleoproteins (RNPs) containing translationally inactive mRNAs from the polyribosomes containing mRNAs that are being translated. Microarray analyses of the two pools of mRNAs showed that the transition between MII oocytes and zygotes at pronuclear stage is accompanied by profound changes in the profile of the transcripts being translated [42]. Gene ontology classification suggested that the ovulated oocyte is engaged in translation of proteins involved in cell homeostasis and signal transduction pathways. By contrast, the zygote appeared to be mainly involved in translation of biosynthetic proteins, possibly required to provide energy and new molecules for the nascent embryo [42]. Translation regulation often occurs by binding of RNA binding proteins (RBPs) to the 3′UTR of the mRNA [43]. Analysis of the 3′UTR of oocyte- or embryo-enriched translated mRNAs revealed the abundance of cytoplasmic polyadenylation elements (CPE), especially in the former group. This observation suggests a central role for the CPE binding proteins (CPEBs) in temporal recruitment of transcripts for translation during the oocyte-embryo transition. Although not formally tested in the cited work, this is reminiscent of the central role played by CPEB1 and CPEB4 in frog oocyte and early embryos, in which CPEB1-dependent translation of CPEB4 allows alternation in translational regulation of different subsets of mRNAs during the two meiotic divisions [44]. Nevertheless, further studies are needed to confirm the role of CPEB proteins in translation control also in mouse oocyte and embryo.

## 5. RNA Decay at the Oocyte-Embryo Transition

Another relevant step in post-transcriptional regulation of gene expression at the onset of development is provided by the degradation of maternal mRNAs. Indeed, ZGA and decay of maternal mRNAs are both essential for the proper development of the embryo, and concur to mould a wider process known as maternal-to-zygotic transition (MZT).

The total mRNA levels decrease by 60% from MII-arrested oocytes to late 1-cell embryos [44], and degradation of the maternal transcripts is almost completed in two-cell embryo, although specific maternal protein synthesis continues until the eight-cell stage [7,45–47]. Recently, it has been proposed that small non coding RNAs, such as microRNAs and endogenous siRNAs, could play a role in RNA homeostasis of oocytes and early embryos. In zebrafish embryos miR-430 promotes deadenylation and degradation of maternal RNAs [48]. In mice, AGO2, the catalytic component of the RNA-induced silencing complex (RISC), is essential for the development beyond the two-cell stage [49]. AGO2 seems to be involved in degradation of some maternal transcripts, hence pointing out a possible involvement of the RNAi machinery in the process of maternal RNA degradation also in mammals [48,50]. Importantly, a recent work documented that the function of microRNAs is suppressed during mouse oocyte maturation and early embryo development [51]. Hence, endogenous siRNAs seems to be the candidate targets of *Dicer* and RISC activity and are likely to be responsible for maternal RNA degradation in zygotes. Since the field of non-coding RNAs is rapidly expanding, it is likely that new insights into their contribution to gene expression regulation in early mammalian development will be unveiled soon.

## 6. Spatial Patterns of Translational Regulation: The Darkest Side of Early Embryogenesis in Mammals

Maternal mRNAs are stored during oogenesis in an inactive state until they are recruited for translation. Their unmasking is accompanied by the elongation of the poly(A) tail, followed by complex changes in protein synthesis patterns [46,52–54]. The distribution of transcripts along a gradient and their specific localization in distinct compartments are devices adopted by the cells to rapidly and finely tune translation of specific mRNAs. Notably, this strategy is especially adopted by highly differentiated and polarized cells, like frog [55] and fly oocytes and embryos [56,57], neurons [58] and fibroblasts [59].

In a wide range of vertebrate and invertebrate embryos, the intracellular localization of mRNAs directs protein synthesis to subcellular domains, thereby establishing embryonic polarity [60]. Translational regulators control the formation of the anterior-posterior axis in the *D. melanogaster* embryo. A remarkable example is represented by the *Bicoid* (*bcd*) mRNA, which is pre-localized at the anterior pole of the oocyte through interaction with sequence-specific RNA-binding proteins (RBPs), and whose translation after fertilization produces a Bcd protein gradient that determines the anterior cell fate [61,62].

Also in *C. elegans* gonads and embryos, RBPs control the spatial and temporal expression of specific genes [63,64]. For instance, the KH protein GLD-1 acts as a translational repressor of several maternal mRNAs by binding to the 3′UTR of target transcripts during gametogenesis [65,66]. Furthermore, GLD-1 promotes the initiation of meiotic development and/or inhibits stem cell proliferation depending on its accumulation pattern [67,68].

When mRNAs are not engaged in translation, they must be saved from the default pathway of degradation. For this reason, RNAs are always bound to proteins in complexes called ribonucleoproteins (RNPs) within the cell. RNPs, in turn, can be organized in multi-molecular structures named RNA granules, which change in shape, components and size in response to environmental cues. In somatic cells two types of granules, the P bodies and the stress granules, have been extensively studied [69,70]. The P bodies contain decapping enzymes, RBPs and components of the microRNA pathways, and have been involved in the control of RNA degradation. Stress granules are transient structures that assemble in the cytoplasm of cells exposed to various stresses. They contain RBPs, ribosomal subunits and mRNAs and are supposed to be sites of storage of transcripts during stress. Similar structures have been characterized also in frog, fly and worm oocytes [56,71–73]. In contrast, scarce information is available on the dynamics of RNA granules during mammalian oogenesis and early development and/or on the structures involved in protection of mRNAs. Furthermore, no conclusive evidence of mRNAs or RNPs distributed along a gradient in mouse oocytes has been reported. It is likely that some sophisticated mechanism(s) exists to protect and regulate the accumulation and release of maternal mRNAs in the mouse zygote. Hopefully advances in technology such as live imaging [74] will make it possible to investigate RNA dynamics *in vivo* and to provide clear-cut answers to these issues.

## 7. Examples of Structures Putatively Involved in Maternal Protein and/or RNA Storage in Mouse Oocytes

It has been recently proposed that the cortex of growing oocytes serves as an mRNA storage compartment, and that it contains a new kind of transient RNA granules related to P-bodies, named sub-cortical RNP domain (SCRD) [75]. Although these data suggest that RNA compartmentalization occurs also in the mouse oocyte and early embryo, they do not clearly explain whether these structures can be considered the equivalent of P bodies or stress granules [49,76].

Some recent papers hypothesize that a structure named cytoplasmic lattice (CPL) could be the site from which maternal factors are released during early embryogenesis, thus playing a role in translational control of maternal transcripts. CPL is a fibrillar matrix that was described many years ago in the oocyte as composed of RNAs and proteinaceous components. This structure persists until the blastocyst stage but its function has remained elusive, even though it was proposed to represent a ribosomal storage site [77–79]. Esposito *et al.* [80] and Yurttas *et al.* [81] demonstrated that *Padi6* is a maternal effect gene required for development beyond the two-cell stage and for CPL formation. *Padi6* null embryos showed impaired transcription and dysregulation of mRNA translation, supporting the hypothesis that the two-cell arrest was due to defective activation of the embryonic genome. A similar role in CPL formation has been described for the maternal-factors MATER [82] and FLOPED [83]. This latter protein contains a single KH (hnRNA K homology) domain, associates with single-strand nucleic acids, and is essential for embryonic development at the MZT [84]. Interestingly, PADI6 protein has also been identified as a component of a structure called Subcortical Maternal Complex (SCMC) [84]. This complex includes four components, partially shared with CPLs: MATER, FLOPED, FILIA (another maternal effect factor [85]) and TLE6 (a putative transcriptional co-repressor). The authors proposed a role for SCMC in the activation of the embryonic genome, hypothesizing that the oocyte cortex could act as a regulatory platform for the organized translation of maternal transcripts [84]. Altogether these findings suggest an involvement of subcellular structures in the mechanisms of translational control adopted by oocytes and early embryo. Nevertheless, whether CPLs and SCMC are competitive or cooperative structures is still an open question.

## 8. RNA-Binding Proteins in Mouse Oocyte and Embryos

The events leading to the development of an organism must be strictly and timely controlled. At the onset of embryogenesis, post-transcriptional mechanisms are the main regulators of gene expression. Although in lower multicellular organisms, these mechanisms rely on the function of RBPs [60–63], much less is known in mammalian oocytes and embryos. Several RBPs are highly abundant in the female gamete, suggesting important roles for these proteins also in mammalian oogenesis and early embryogenesis. The CPEB1 protein is one of the best characterized regulators of mRNA translation initiation, which can act as repressor or activator depending on its partners and phosphorylation status [86]. Importantly, knockdown of CPEB1 expression during oogenesis impairs oocyte maturation and fertility, but the relevant mRNA targets in mouse oocytes are still unknown. MSY2 is another RBP required for the formation of developmentally competent oocytes, whose absence impairs fertilization [87]. However, its exact role in post-transcriptional regulation of mRNAs in mouse oocytes is currently unknown. The mammalian homologues of the *C. elegans* GLD-1 protein, Sam68, SLM-1 and SLM-2, are also potential candidates for a role in early embryogenesis [88]. In particular, Sam68 is highly expressed in oocytes [89] and spermatocytes [90], and it plays crucial functions in male [90] and female fertility [91]. Nevertheless, its activity in oocytes seems to be redundant [89], possibly due to expression of the highly homologous SLM-1 in the female gamete [89]. Altogether, these observations suggest that more studies are required to determine the role of specific proteins in post-transcriptional regulation of gene expression in the early mouse embryo.

## 9. Conclusions

Fertilization is the most important step for conservation of the species. The fusion of two gametes is required to generate a new organism in higher eukaryotes, and assortment of maternal and paternal chromosomes ensures diversity of the offspring. Importantly, the first steps of embryo development occur in the absence of transcription of the embryonic genome, and rely on post-transcriptional regulation of maternally-inherited mRNAs. Thus, appropriate control of degradation or translation of maternal transcripts is likely required to drive the onset of embryonic genome activation. It is predictable that full understanding of post-transcriptional regulation of gene expression in early embryos will shed light on the molecular mechanisms that regulate reprogramming of the embryonic genome. This knowledge might open new avenues also for the reprogramming of stem cells to be used in regenerative medicine or for the treatment of genetic diseases. On the other hand, such studies might also aid in improving protocols for *in vitro* culturing of mammalian oocytes and embryos to be used for assisted reproduction. In mammals, the paucity of the starting material has strongly limited classical biochemical approaches used to elucidate the molecular mechanisms of post-transcriptional control of gene expression. Nevertheless, the amazing improvement of *in vivo* techniques for the analysis of protein and RNA functions, coupled with the development of high-sensitivity molecular tools, is rapidly filling the gap of our knowledge on the regulation of post-transcriptional control of gene expression also in mammalian oocytes. Thus, although much still remains to be discovered in order to build up a comprehensive vision of the mechanisms driving the onset of mammalian development, it is foreseen that the next years will witness a bloom of studies in this fascinating field of biology.

## Abbreviations

MII: second meiotic division
ZGA: zygotic genome activation
HOX: homeobox
NPM2: nucleoplasmin2
BRG1: BRM/SWI2-related gene 1
SWI/SNF: Switch/Sucrose Non Fermentable
BRWD1: bromodomain and WD repeat domain containing 1
*Dppa3*: developmental pluripotency-associated 3
DNMT 1: DNA methyltransferase DNMT1
ZFP57: zinc-finger protein 57
ZAR1: zygote arrest 1
RNPs: ribonucleoproteins
UTR: untranslated region
RBPs: RNA binding proteins
CPE: cytoplasmic polyadenylation elements
CPE: binding proteins: CPEBs
MZT: maternal-to-zygotic transition
AGO2: argonaute 2
RISC: RNA-induced silencing complex
*bcd*: *Bicoid*
GLD1: defective in germ line development 1
SCRD: sub-cortical RNP domain
CPL: cytoplasmic lattice
KH: hnRNA K homology
SCMC: Subcortical Maternal Complex
MSY2: germ-cell-specific Y-box-binding protein
Sam68: src-associated in mitosis
SLM-1: sam68-like mammalian protein
SLM-2: sam68-like mammalian protein
